# Effect of 3% Hypertonic Saline Resuscitation on Lactate Clearance and Its Comparison With 0.9% Normal Saline in Traumatic Injury Patients: A Prospective Randomized Control Trial

**DOI:** 10.7759/cureus.38836

**Published:** 2023-05-10

**Authors:** Kavita Meena, Suprabhat Gautam, Tenzin Kyizom, Rajesh K Meena, Aditya P Nayak, Shashi Prakash

**Affiliations:** 1 Anaesthesiology, Banaras Hindu University, Varanasi, IND; 2 Anaesthesiology, Sh. Guru Ram Rai Institute of Medical and Health Sciences, Dehradun, IND

**Keywords:** traumatic injury, normal saline, lactate clearance, shock, fluid resuscitation, hypertonic sodium chloride

## Abstract

Background: Fluid resuscitation with normal saline (NS) can aggravate lactate production. The objective of this study was to evaluate the efficacy of small-volume resuscitation using 3% hypertonic sodium chloride (HS) and its comparison with NS in trauma patients. The primary endpoint was an increase in lactate clearance after 1 hr of fluid resuscitation. The secondary endpoint was the incidence of hemodynamic stability, the volume of transfusion, correction of metabolic acidosis, and complications such as fluid overload and abnormal serum sodium levels.

Materials and methods: It was a prospective, randomized, single-blind study. The study was conducted on 60 patients who arrived at the trauma center for emergency operative intervention. Inclusion criteria for patient selection were trauma victims of age more than 18 years and the requirement of emergency operative intervention for trauma except for traumatic brain injury. Patients were divided into two groups: Group HS (hypertonic saline) and Group NS (normal saline). Patients were resuscitated with either 3% HS (4ml/kg) or 0.9% NS (20ml/kg).

Results: The HS group had higher lactate clearance at 1 hour compared to the NS group, and this difference was statistically significant with a p-value of <0.001. When hemodynamic parameters were compared at 30 and 60 minutes after resuscitation, the HS group had a significantly lower heart rate (p<0.05 at 30 minutes and <0.001 at 60 minutes, respectively), a higher mean arterial pressure at 60 minutes (p<0.001), a higher pH at 60 minutes (p< 0.05), and a higher bicarbonate concentration at 60 minutes (p<0.05). The HS and NS groups had significant differences in serum sodium levels at 60 minutes (p<0.001).

Conclusions: Resuscitation with 3% hypertonic saline improved lactate clearance. Lower volumes of fluid infusion for resuscitation achieved better hemodynamic stability and correction of metabolic acidosis in the hypertonic saline group. Our study shows that hypertonic saline can be a promising fluid for small-volume resuscitation in trauma patients with compensated mild to moderate shock.

## Introduction

Trauma is a global, social, and economic burden, accounting for a third of the overall causes of mortality. Severe trauma is experienced in all age groups; however, the most affected group is the younger age group (< 45 years of age). In total, 80% of the deaths happen within 48 hours, with hemorrhage being one of the leading causes of death [1]. Hemorrhage is preventable and can be controlled by effective fluid resuscitation and damage-control surgery. However, fluid resuscitation in patients with trauma has always been controversial. The guidelines for fluid selection are continuously evolving, with no consensus on the choice of fluid. Fluid resuscitation also leads to a large volume of fluid being infused into the patient, causing dilutional coagulopathy, which further deteriorates the patient’s condition. So, healthcare professionals are focusing on low-volume resuscitation. The efficacy of a resuscitation strategy can be assessed clinically by serial monitoring of laboratory parameters such as hemoglobin, blood gases, lactate, and hemostasis. Recently, lactate clearance has emerged as an important marker of fluid resuscitation in patients with trauma. Serum lactate levels correlate with anaerobic metabolism, and the serial measurement of serum lactate levels shows effective fluid resuscitation, which can guide the evaluation and change in the resuscitation strategy [2]. The normalization of serum lactate with aggressive treatment within 24 hours of an incident has been shown to have a favorable outcome, according to several studies with different fluid resuscitation approaches.

This study aims to compare lactate clearance in patients suffering from trauma with fluid resuscitation by using 3% hypertonic saline and 0.9% normal saline. We hypothesized that low-volume resuscitation using 3% hypertonic saline would show improved outcomes.

## Materials and methods

Sample-size determination and statistical analysis 

To calculate the sample size for measuring and comparing the mean lactate clearance of the two groups, the sample sizes were determined based on the formula that examines the differences in mean, with significance levels α = 5% (Zα = 1.65), ß = 20% (Zß = 0.84), and SD = 5.8. We included 30 patients in each group. The impact of the solution on lactate clearance levels over time in each group was analyzed. The data were presented as the mean and standard deviation. The demographic data of the patients were studied for each group and presented in frequency and percentages. The mean comparison between the two groups was done using the nonparametric student’s unpaired t-test. Nominal categorical data between the groups was compared using the chi-square test or Fisher’s exact test, as appropriate. A p-value of less than 0.05 was considered statistically significant.

Methods: This was a prospective, randomized, case-control clinical study conducted at the triage of a level I trauma center from January 2022 to June 2022. Approval from the institutional ethical committee was obtained, and the trial was registered with the clinical trial registry (REF/2022/01/050238). The randomization of 60 patients was done using computer-generated random number tables. The inclusion criteria for patient selection were 18 years of age or older, victims of road accidents, and patients posted for emergency orthopedic and abdominal surgeries. Patients with traumatic brain injury, comorbidities, bleeding diathesis, allergies to colloids or other drugs, and serum electrolyte abnormalities who required inotropic support at the time of presentation were excluded from the study. Written informed consent from the patients or their attendants was obtained. 

The patients who fulfilled the inclusion criteria were divided into two groups: group NS (normal saline, n = 30) and group HS (hypertonic saline, n = 30); group allocation was done using the sealed envelope technique. When a patient was received in the triage area of the trauma center, the primary survey and fluid resuscitation were started. In group NS, the patients received 0.9% NS at the rate of 20 ml/kg for 30 minutes, and in group HS, the patients received 3% HS at the rate of 4 mL/kg for 30 minutes. The enrolment and resuscitation of patients were performed by the authors and four trained senior residents. The intervention period for the study ran for the first hour of treatment, and serial monitoring and lab tests were done during this period. The outcomes, complications, adverse events, and concomitant treatment given to the patients were recorded. If shock persisted, inotropes and/or catecholamines were commenced, and blood transfusion was started as required. 

Blood samples were drawn from the patients for the measurement of baseline lactate levels simultaneously with fluid resuscitation. The samples were drawn at the time of intravenous cannulation, and investigations such as a complete hemogram, blood grouping, Rh typing, and random blood sugar were conducted. An electrocardiogram (for patients over 40 years of age) and chest X-ray were also done. The Glasgow coma score was also assessed. Arterial blood gas analysis (ABG), serum electrolytes, serum urea/creatinine, and lactate levels were measured at baseline at the time of patient presentation. Serial monitoring of vital parameters was done at 0, 15, 30, and 60 mins. The ABG parameters were recorded 30 and 60 min after the completion of the calculated dose infused with fluids. Apart from the above-mentioned parameters, the urine output of the patients was also recorded in the intraoperative period, and the patients were observed for any reactions or complications. After 60 min, each patient received a blood transfusion. 

The lactate clearance was calculated as follows: 

1-hour lactate clearance (%) = (0-hour lactate - 1-hour lactate) / 0-hour lactate × 100

The primary endpoint was the increase in lactate clearance after an hour of fluid resuscitation. The secondary endpoint was the hemodynamic parameters, the volume of transfusion, and correction of metabolic acidosis and complications such as fluid overload and serum sodium level.

## Results

**Figure 1:**
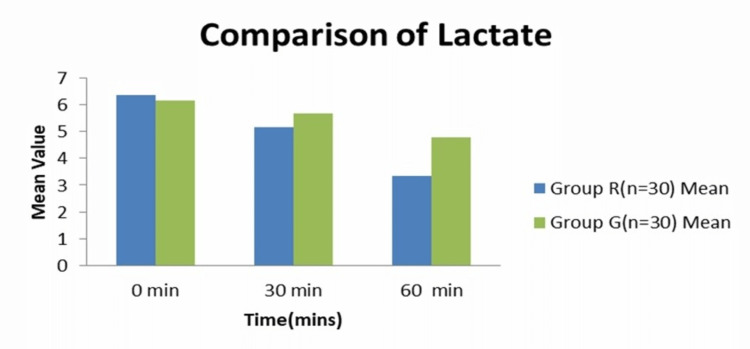
Lactate concentration on ABG at time intervals 0, 30, 60 minutes (mmol/l)

The demographic and clinical profile of the patient (Table 1) were comparable at baseline, with no statistically significant difference. The mean lactate concentration (Figure 1) on ABG in group R at 0 min was 6.37 mmol/l; at 30 min (at the end of fluid transfusion), it was 5.15± 0.86 mmol/l, and at 60 min, it was 3.35 0.72 mmol/l. In Group G, the mean lactate concentration on ABG in Group R at 0 min was 6.16 0.75 mmol/l; at 30 min (at the end of fluid transfusion), it was 5.68± 0.81 mmol/l; and at 60 min, it was 4.79±0.72 mmol/l. The difference in the mean was calculated using the Student’s Unpaired 't'-test. The p-values obtained at 0 min was 0.303, which is statistically insignificant. The p-value obtained at 30 min and 60 min were 0.000, which is statistically significant. The mean lactate clearance (Table 2) at one hour after fluid resuscitation in Group HS was 47.05 ± 11.24, and in Group NS, it was 22.13 ± 6.89. The difference in the mean was calculated using Student’s Unpaired 't'-test. The p-value obtained was 0.000 (< 0.05), which was statistically significant. Baseline hemodynamic and ABG parameters (Table 3) were not significantly different between groups, but the mean heart rate and mean arterial pressure at 30 min and 60 min were statistically significant (p<0.05). The volume of transfused fluid (Table 4) was based on the weight of both groups, and this volume was given within 30 min. There was a significant (p-value<0.001) increase in sodium level at the end of 1 hour after fluid resuscitation compared to the baseline and at 30 min, but none of the patients in either group developed hypernatremia or renal failure. The mean potassium concentration on ABG at 30 min was 4.35 ±0.30 mmol/l, and at 60 min, it was 4.49 ± 0.28 mmol/l in group HS. Serum electrolyte concentration on ABG (Table 5) during fluid resuscitation was measured. There was a significant (p-value <0.001) increase in the sodium concentration at the end of one hour after fluid resuscitation compared to baseline and at 30 min, but none of the patients developed hypernatremia or renal failure in either group. The potassium concentration on ABG at 30 min was 4.35 ± 0.30 mmol/l, and at 60 min, it was 4.49 ± 0.43 in group HS. The mean potassium ion concentration at 30 min was 4.36 ± 0.28 mmol/l, and at 60 min, it was 4.46± 0.41 mmol/l. The difference in the mean was calculated using an unpaired 't'-test. The p-values obtained at 30 min and 60 min were 0.865 and 0.767, respectively, which were statistically not significant. The mean chloride concentration on ABG in group HS at 30 min was 102.77±3.59 mmol/l, and at 60 min, it was 105.49± 1.95 mmol/l. In group NS, the mean chloride ion on ABG at 30 min was 101.72 ±3.16 mmol/l, and at 60 min, it was 105.37±1.98 mmol/l. The difference in the mean was calculated using an unpaired 't'-test. The p-values obtained at 30 min and 60 min were 0.234 and 0.814, respectively, which were statistically not significant. The mean urine output at 60 min in group HS was 148±21.39 ml, and in group NS, it was 151± 22.10 ml. The mean difference was calculated using the student unpaired 't'-test. The p-value obtained was 0.590, which was statistically significant.

**Table 1:** Demographic and clinical profile of the patients in two groups Data expressed as mean and standard deviation. M/F: male/female, mg/dl: milligram per deciliter, bpm: beats per minute, mmHg: millimeters of mercury, SPO2: peripheral saturation of oxygen, mmoL: millimole per liter

Parameters	HS (N=30)	NS (N=30)	p-value
Age (years)	42.60±10.94	42.80±10.59	0.942
Gender M/F	16/14	18/12	0.609
Weight (Kilograms)	59.10±5.90	59.06±3.87	0.979
Serum Urea (mg/dl)	43.90±4.65	43.10±4.44	0.490
Serum creatinine (mg/dl)	1.20±0.14	1.23±0.13	0.410
Heart rate (bpm)	125.66±5.06	124.53±4.41	0.359
Mean Arterial pressure (mmHg)	63.10±2.17	63.43±2.14	0.212
Systolic Blood Pressure (mmHg)	77.96±3.81	77.86±4.56	0.926
Diastolic Blood Pressure (mmHg)	57.76±3.01	56.83±2.88	0.225
SPO2	99.13±0.89	98.96±0.96	0.490
pH	7.27±0.02	7.28±0.02	0.505
Na (mmol/l)	131.97±1.48	131.70±1.22	0.439
K (mmol/l)	4.42±0.33	4.46±0.31	0.616
Cl (mmol/l)	98.81±3.48	97.71±2.74	0.392
HCO3 (mmol/l)	20.65±1.57	20.79±1.17	0.698
Ca (mmol/l)	1.02±0.06	1.03±0.06	0.392
Lactate (mmol/l)	6.37±0.78	6.16±0.75	0.303

**Table 2:** Comparison of lactate clearance at the end of 1 hour after fluid resuscitation in two groups

Parameter	HS(N=30)	NS(N=30)	p-value
1 hour lactate clearance	47.05±11.24	22.13±6.89	<0.001
Data expressed as mean and standard deviation.			

**Table 3:** Impact of fluid resuscitation on hemodynamic and ABG parameters Data expressed as mean and standard deviation. Bpm: beats per minute, mmHg: millimeters of mercury mg/dl; milligram per deciliter

Parameter	HS(N=30)	NS(N=30)	p-value
HR-30 min (bpm)	113.60±3.85	116.40±5.49	0.026
HR-60 min (bpm)	103.83±4.37	108±5.93	0.001
MAP-30 min (mmHg)	67.80±2.81	66±2.92	0.031
MAP-60 min (mmHg)	77.90±3.24	73±2.87	<0.001
SBP-30 min (mmHg)	81.90±5.18	80.23±4.58	0.192
SBP-60 min (mmHg)	92.73±4.63	88.63±4.35	0.001
DBP-30 min (mmHg)	61.26±3.33	59.80±4.38	0.150
DBP-60 min (mmHg)	70.96±3.92	66.63±4.45	0.001
pH-30 min	7.28±0.02	7.28±0.01	0.838
pH-60 min	7.31±0.01	7.29±0.01	0.011
Na-30 min (mmol/l)	133.22±1.53	132.61±1.07	0.082
Na-60 min (mmol/l)	136±1.85	134±0.96	<0.001
HCO3-30 min (mmol/l)	20.89±1.25	20.91±0.66	0.938
HCO3-60 min (mmol/l)	22.01±0.85	21.57±0.58	0.025

**Table 4:** Total volume of fluid transfused in Group HS and Group NS Values expressed in mean and standard deviation. ml: millilitre

Group	HS (N=30)	NS (N=30)	P-value
Volume transfused (ml)	354.80±35.22	1772±116.31	<0.001

**Table 5:** Serum electrolyte concentrations on ABG during fluid resuscitation Data expressed as mean and standard deviation. mmol/L, ml: milliliter

Parameters	HS (N=30)	NS (N=30)	p- value
Na-30 min (mmol/L)	133.22±1.53	132.61±1.07	0.082
Na-60 min (mmol/l)	136.63±1.85	134.81±0.96	<0.001
K-30 min (mmol/L)	4.35±0.30	4.36±0.28	0.616
K-60 min (mmol/L)	4.49±0.43	4.46±0.41	0.767
Cl-30 min (mmol/L)	102.77±3.59	101.72±3.16	0.234
Cl-60 min (mmol/L)	105.49±1.95	105.37±1.98	0.814
Urine output-60 min (ml)	148±21.39	151±22.10	0.590

## Discussion

Hemorrhage in trauma victims is a major cause of morbidity and mortality [3]. Hypovolemia leads to tissue hypoxia and lactate production. The management of hemorrhage requires prompt resuscitation using fluids and blood products, but large-volume resuscitation further aggravates blood loss due to hypothermia and dilutional coagulopathy. A large volume of fluid resuscitation may also lead to abdominal compartment syndrome and lung injury. 

Recent studies have focused on low-volume resuscitation. Hypertonic saline (HS; 7.5% saline with or without colloids) has long been considered a fluid of interest for the treatment of patients with trauma. Potential benefits of HS include the restoration of intravascular volume with the administration of a small volume due to its osmotic effect that shifts fluid from the intracellular space to the extracellular space, reduction of intracranial pressure in TBI, and modulation of the inflammatory response. 

The effectiveness of fluid resuscitation can be monitored using lactate clearance. This was the first study assessing the effects of 3% HS on lactate clearance in patients with trauma. In this study, a significant decrease in serum lactate concentration occurred an hour after resuscitation in the HS group, whereas in the NS group, the serum lactate tended to increase. One-hour lactate clearance in the HS group was significantly higher than that in the NS group (p < 0.001). A study by Guo-chao Zhu revealed that at six hours after ICU admission, the lactate clearance rate in the 7.5% HS+HES (hydroxyethyl starch) group was significantly higher than that in the HES and RL groups [4]. Nevin Chandra Junarsa revealed that the HSL (hyperosmolar sodium lactate) group had significantly higher 1-hour and 6-hour lactate clearance than the NS group (p < 0.05 and p < 0.01, respectively) [5]. The results of lactate clearance in our study are not in accordance with those of the study conducted by Luis Horacio Atehortúa-Lópezain in 2017 [6], and this difference can be attributed to the patient profile. In our study, we included patients with trauma who suffered from acute blood loss and had already presented to triage in a decompensated state, whereas in the above-mentioned study, the selected patients had been admitted for elective cardiac surgery.

HS improved the microcirculation and organ perfusion more than NS. Thus, the infusion of 3% HS resulted in increased pH, bicarbonate concentration, and sodium concentration at 60 min as compared with 0.9% NaCl. This shows that a lower volume of 3% NaCl gives a better outcome for the correction of metabolic acidosis when compared with 0.9% NaCl, which is usually present in trauma and hemorrhage. However, due to the high concentration of sodium in 3% HS, a significant rise in sodium concentration was observed, but none of the patients developed hypernatremia in either group. This also depicts that the infusion of 3% NaCl at 4 ml/kg increases the sodium concentration after the infusion but can be given safely without the risk of hypernatremia. There are no similar studies for reference in the literature, but the study conducted by Luis Horacio Atehortúa-Lópezain in 2017 on cardiac patients found no significant difference in lactate clearance when 7.5% HS was used compared with 0.9% saline. That study also observed no significant increase in the sodium concentration, even in the 10 AKI patients present in their study; we found no hypernatremia as well in our study. In the study performed by Duburcq T [7], arterial pH and bicarbonate levels decreased over time in the NS group. On the other hand, these variable levels increased over time in the hypertonic groups without any differences between the HSB (hypertonic sodium bicarbonate) and HSL (hypertonic sodium lactate group) groups. Compared with the NS group, pH and bicarbonate levels were significantly higher in the HSB and HSL groups from 120 to 300 minutes. Nevin Chandra Junarsa revealed that the sodium level before resuscitation was low in both groups, although this was not statistically significant. There was an increase in the sodium level by this time, but the increase was not significantly different between the groups. Only one subject in the HSL group experienced hypernatremia after fluid resuscitation (150 mEq/L), with no clinical manifestation. Kati Ja¨rvela revealed that hypertonic saline infusion in doses of 1.6 ml/kg and 4 ml/kg elevates sodium concentration and chloride levels, but no notable changes are observed in the potassium concentration [8].

HS keeps the patient hemodynamically stable compared with NS. In our study, 3% NaCl caused a decrease in heart rate and an increase in MAP as compared with 0.9% NaCl after 30 min. This shows that 3% NaCl infusion offers better hemodynamic stability at low volume at the same time. In both groups, mean systolic and diastolic blood pressure increased, but the level of increase was found to be statistically significant only at 60 min. A study by Zhu GC revealed that at 6 hours after ICU admission, MAP in the HS+HES group was significantly higher than that in the HES and RL groups, but MAP was not significant at 24 hours. Another study by Kati Ja¨rvela revealed that HS infusion at 1.6 ml/kg and 4 ml/kg has positive hemodynamic effects. Both MAP and CI increased at higher levels for approximately an hour. However, no difference in heart rate was observed between the study groups [8]. Sahar M. Talaat also revealed that two patients (10%) in the HS group, six patients (30%) in the HES group, and 13 patients (65%) in the NS group experienced hypotension, showing that the incidence of hypotension was significantly lower in the former two groups [9]. The total dose of ephedrine required by the HS and HES groups was significantly lower compared with that required by the NS group. Moreover, the heart rate showed no significant differences among the three groups throughout the procedure.

We found a significant difference in the volume of fluid transfused between the groups. Hypertonic crystalloids such as HS produce a high osmotic gradient, which increases the plasma volume three-to-four times when using small-volume resuscitation. Small-volume resuscitation using hypertonic HSL could cause hypernatremia, but none of the patients in this study experienced it after fluid resuscitation with HS at 4 ml/kg. Small-volume resuscitation with HS has the advantage of improving microcirculatory hemodynamics faster, modulating the immune response, and preventing reperfusion injury and oxidative and nitrosative stress due to RNS and ROS production. This advantage has protective effects for vital organs from hypoxia, ischemia, and reperfusion. 

This study also has some limitations. First, the sample size was relatively small. Moreover, the study was only conducted for one hour. Serial monitoring for up to 24 hours and the exact dose and concentration of HS should be further studied in patients with trauma. A multicenter study with a larger sample size and adequate fluid monitoring is needed.

## Conclusions

Fluid resuscitation with HS showed increased lactate clearance compared with NS. Resuscitation with HS provides small-volume resuscitation, avoiding fluid overload. Hemodynamic parameters are better maintained in terms of decreasing heart rate, increasing MAP (> 65 mm Hg), and metabolic acidosis correction in terms of pH and bicarbonate concentration. Hyperosmolar sodium is a promising fluid for resuscitation in patients with trauma who are at risk of fluid overload.
